# Regulatory T Cell Metabolism in the Hepatic Microenvironment

**DOI:** 10.3389/fimmu.2017.01889

**Published:** 2018-01-08

**Authors:** Rebecca Ellen Wawman, Helen Bartlett, Ye Htun Oo

**Affiliations:** ^1^Centre for Liver Research, National Institute of Health Research Birmingham Biomedical Research Centre, Institute of Immunology and Immunotherapy, University of Birmingham, Birmingham, United Kingdom; ^2^Faculty of Health and Life Sciences, School of Life Sciences, Coventry University, Coventry, United Kingdom; ^3^University Hospitals Birmingham NHS Foundation Trust, Birmingham, United Kingdom

**Keywords:** regulatory T cells, microenvironment, liver, Treg plasticity, function, immunometabolism, good manufacturing practice Treg, cell therapy

## Abstract

Thymic-derived naturally occurring regulatory T cells (tTreg) are crucial for maintaining peripheral immune homeostasis. They play a crucial role in preventing autoimmunity and maintaining organ transplant without requiring immunosuppression. Cellular metabolism has recently emerged as an important regulator of adaptive immune cell balance between Treg and effector T cells. While the metabolic requirements of conventional T cells are increasingly understood, the role of Treg cellular metabolism is less clear. The continuous exposure of metabolites and nutrients to the human liver *via* the portal blood flow influences the lineage fitness, function, proliferation, migration, and survival of Treg cells. As cellular metabolism has an impact on its function, it is crucial to understand the metabolic pathways wiring in regulatory T cells. Currently, there are ongoing early phase clinical trials with polyclonal and antigen-specific good manufacturing practice (GMP) Treg therapy to treat autoimmune diseases and organ transplantation. Thus, enhancing immunometabolic pathways of Treg by translational approach with existing or new drugs would utilize Treg cells to their full potential for effective cellular therapy.

## Regulatory T Cells and Peripheral Self-Tolerance

**Figure 1:**
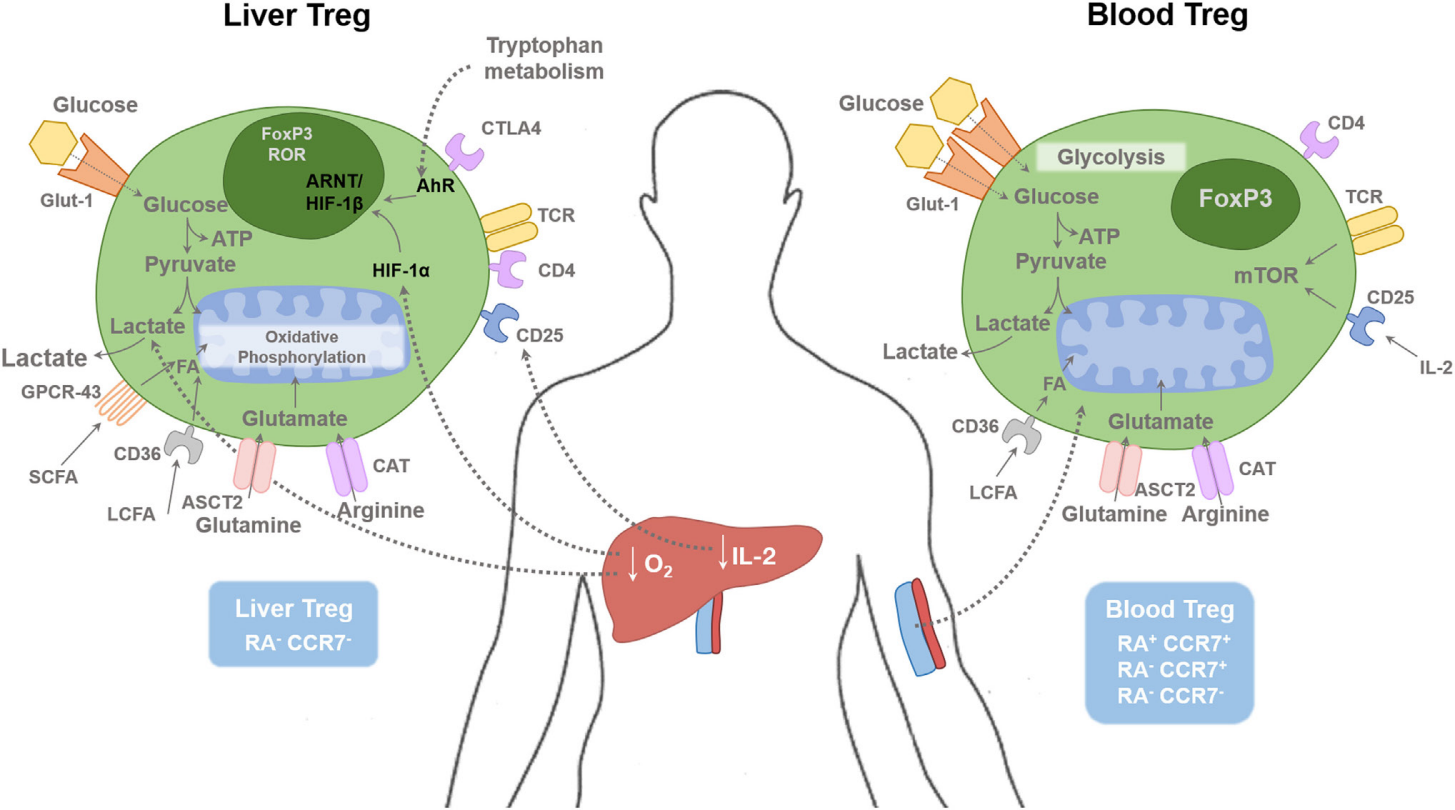
Regulatory T cells in tissue and blood compartments. The human liver is a hypoxic environment as the majority of blood flow is from the portal venous system. This leads to hypoxic induced factor 1-α (HIF-1α) activation, which subsequently enhances FoxP3 expression along with Th17 differentiation. Hypoxia leads to anaerobic glycolysis and extracellular lactic acid accumulation. Short-chain fatty acids (SCFAs) bind to the receptor GPCR43; long-chain fatty acids (LCFAs) bind to CD36, glutamine binds to ASCT2, and arginine binds to CAT2. Glucose transporter-1 (Glut-1) is poorly expressed on Treg cells compared with effector T cells. Liver Treg cells are mainly of an effector memory phenotype (RA^−^CCR7^−^). There is only minimal level of IL-2 present in the human liver compared with the blood, which restrict hepatic Treg function. Blood Treg-cell subsets are composed of effector memory (RA^−^CCR7^−^), central memory (RA^−^CCR7^+^), and naive (RA^+^CCR7^+^) phenotype. HIF-1α, Hypoxia-inducible factor 1α; HIF-1β, Hypoxia-inducible factor-1β; AhR, aryl hydrocarbon receptor; FA, Fatty acids; ARNT, Aryl Hydrocarbon Receptor Nuclear Translocator; mTOR, mammalian target of rapamycin; SCFA, short chain fatty acid; LCFA, long chain fatty acid; ASCT2, Alanine, serine, cysteine-preferring transporter 2; CAT, Cationic amino acid transporter; GPCR, G protein–coupled receptor.

Regulatory T (Treg) cells are a subset of CD4^+^ T cells that maintain peripheral immune homeostasis by suppressing a range of untoward immune responses thus maintaining the balance between immune activation and tolerance (1). Sakaguchi and colleagues first reported Treg cells in 1995 *via* adoptive transfer studies, which demonstrated the subset of CD4^+^ T cells expressing the interleukin-2 (IL-2) receptor alpha chain, CD25, preventing autoimmune diseases (2) (Figure 1). Around 5–10% of CD4^+^ T cells are CD25^+^, they are able to maintain peripheral immunologic self-tolerance by suppressing self-reactive lymphocytes (1, 2). Subsequently, Seddiki (3) and Liu (4) reported that low level expression of the IL-7 receptor, CD127, inversely correlated with FoxP3 expression and Treg cell’s suppressive function due to the repressor function of FoxP3. FoxP3 is a master transcription factor and regulator of Treg phenotype and function (5). Mutations in the FoxP3 gene cause defective development of CD4^+^CD25^+^ Treg cells, leading to IPEX syndrome (immunodysregulation, polyendocrinopathy, enteropathy, X-linked genetic trait) (6). Lymphoproliferation and multiorgan autoimmunity in scurfy mutant mice is caused by the absence of FoxP3 (7). FoxP3 is regulated by conserved non-coding DNA sequences (CNS) 1–3. CNS2 is required for FoxP3 expression in the dividing Treg cell and CNS3 controls *de novo* Foxp3 expression and thymic Treg-cell differentiation (8). Therefore, Treg cells are currently defined as CD4^+^CD25^high^CD127^low/−^FoxP3^+^ cells.

Treg cells are essential for maintaining peripheral tolerance by controlling autoreactive T cells, which escape negative selection in the thymus (9). They can be broadly divided into two types; thymic-derived Treg (tTreg) cells and peripheral Treg (pTreg) cells (10). Strong T cell receptor (TCR) signaling with CD28 co-stimulation, just below the threshold for negative selection, promotes tTreg lineage commitment in the thymus (11). pTreg cells are generated in the periphery from populations of mature T cells under certain antigenic stimulating conditions; persistent weak TCR stimulation along with IL-2, transforming growth factor-β (TGF-β) or retinoic acid (RA) (12, 13). The DNA in tTregs is demethylated in the Treg-specific demethylated region (TSDR) in the FoxP3 enhancer, whereas the TSDR of pTregs is only partially demethylated (14). Although both tTreg and pTreg are difficult to distinguish phenotypically, both are thought to play an essential role in immune regulation (15), with tTreg cells controlling reactivity toward self-antigens and pTreg cells controlling responses to antigen exposure in the periphery. Treg cells require IL-2 to maintain their function and survival. Because Treg cells do not make IL-2, they are dependent on IL-2 derived from other T cells (16). Treg cells are highly sensitive to IL-2, due to their constitutively high expression of CD25 and amplified intracellular signal transduction downstream of the IL-2 receptor, phosphorylation of STAT5 to upregualte essential Treg functional gene such as CD25, FoxP3, and cytotoxic T lymphocyte-associated antigen 4 (CTLA-4) (17). Treg cells can therefore compete with conventional T cells for IL-2 as a mechanism to prevent unwanted immune responses (17).

Treg conduct their suppressive function *via* multiple mechanisms throughout different compartments of the body. Treg are therefore also equipped with various functional markers. In the context of liver disease, they constitutively express CTLA-4 (16, 18, 19), ectonucleoside triphosphate diphosphohydrolase 1, CD39 (16, 20), and the intracellular immunosuppressive cytokine, IL-10 (16, 21).

Cytotoxic T lymphocyte-associated antigen 4 is a target gene of FoxP3 (22), and activation of Treg results in upregulation of CTLA-4; its deficiency in mice leads to fatal lymphoproliferation and multiorgan lymphocyte infiltration (23, 24). CTLA-4 binds the ligands CD80 and CD86 on antigen-presenting cells (APCs) such as dendritic cells (DCs). Its mechanism has been reported as removal of these ligands from APC cell surface by trans-endocytosis, which subsequently prevents the effective activation of naïve CD4^+^ T cells by APCs (25). CTLA-4-CD80/CD86 binding leads to upregulation of indoleamine 2,3-dioxygenase (IDO), which catabolizes tryptophan (Trp) into immunosuppressive kynurenines (26). CD39 on both human and murine Treg exert their function *via* generation of adenosine by the breakdown of adenosine triphosphate (ATP) and other extracellular nucleotides, which then bind to adenosine 2A receptors expressed on effector T cells causing a rise in intracellular cyclic adenosine monophosphate, thus inhibiting proliferation of effector T cells (27).

## Intrahepatic Microenvironment

The phenotype and function of Treg cells in circulatory and intrahepatic compartments is different as the intrahepatic microenvironment is hypoxic and enriched with cytokines and metabolic products (1) (Figure 1). Intrahepatic Treg cells respond to (i) engagement of the TCR with MHC Class II on APCs, (ii) the binding of CD28/CTLA-4 on cells with CD80/86 on APCs, and (iii) the influence of cytokines from APC for their activation, survival, and differentiation (28, 29). We reported that the intrahepatic microenvironment is highly enriched with the pro-inflammatory cytokines IL-1β, IL-6, and IL-12 (16) from hepatic DCs but lacks the crucial Treg cell survival cytokine; IL-2 (19). With the recent advances in research into the metabolism of individual immune cell including T cells, it is now realized that differentiation, survival, and function of Treg cells depends not only on TCR, co-stimulatory and cytokine signals but also on other signals in the environment, specifically the local milieu of oxygen, metabolites, and catabolites (30).

## Metabolic Influence on Plasticity, Function, Survival, and Migration of Treg Cells

**Figure 2:**
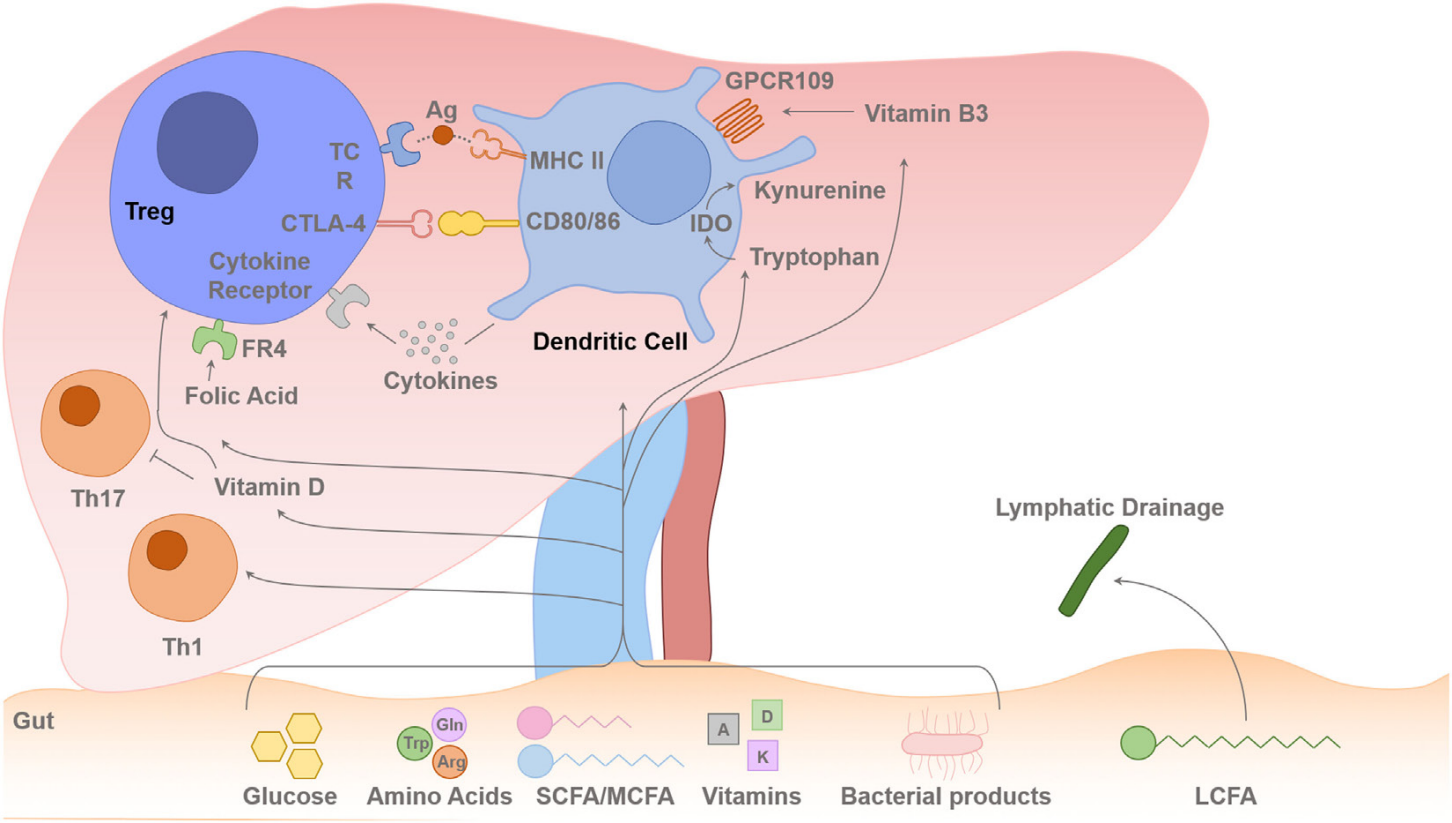
Gut–liver axis and metabolites flux toward the liver. Intrahepatic T cell (including Treg cells) lineage fitness, function, survival, and proliferation are influenced by signals from the antigen presenting dendritic cells (DCs) and cytokines. The T cell receptor (TCR) binding to the foreign antigen–MHC II complex and co-stimulation provided by CD80 and CD86 binding to the T cells CD28/CTLA-4 induces T cell activation or inhibition of its function. Cytokines secreted by the DCs determine the differentiation pathway of the activated CD4 T cell to different Th1/Th2/Th17 lineages or Treg-cell survival with contact-dependent mechanism with DCs CD80/86. IL-2 is secreted in the liver by activated T effector cells, and this is required for Treg-cell survival and suppressive function. Dietary SCFAs (acetate, propionic acid, and butyrate) and MCFAs arrive to the liver *via* the portal vein, but LCFAs are absorbed *via* intestinal lymphatics and drain back into the systemic circulation *via* the thoracic duct. The amino acids, Glu and Arg, and glucose are also absorbed *via* the portal venous system towards the liver. There is always a certain degree of gut leakiness and bacterial product such as lipopolysaccharide reaches to the liver, and they rapidly undergo phagocytosis by hepatic sinusoidal Kupffer cells, which function as a sinusoidal firewall of the liver (32). The liver is enriched with fat-soluble vitamins such as vitamins A and D. DCs express immunosuppressive enzyme indoleamine 2,3-dioxygenase (IDO), which transform Trp into kynurenine, which is then metabolized into other catabolites through the action of enzymes within the kynurenine pathway (26, 33, 34). Trp-derived catabolites can mediate the tolerogenic effects of IDO by inducing apoptosis of activated but not resting T cells (35). Folic acid binds to folic acid receptor (FR4) on the Treg cell and Niacin (vitamin B3) interacts with GPCR109 on DCs. Glu, glutamine; Trp, tryptophan; Arg, arginine; SCFA, short-chain fatty acid; MCFA, median chain fatty acid; LCFA, long-chain fatty acid.

The human liver is uniquely situated to receive a blood supply from the portal venous system, which is enriched with metabolites and nutrients. Human liver has a dual blood supply, deriving 70–80% of its blood, rich in nutrients, from the portal vein and the other 20–30%, rich in oxygen, from the hepatic artery (31). Thus, Treg and T effector cells reside in the hepatic microenvironment with continuous exposure to metabolic signals (Figure 2). Resting T cells require little energy generation or expenditure; however, upon activation, their energy needs increase substantially, and they utilize glucose, amino acids, and fatty acids (FAs) to meet this demand. Metabolic effects on Treg cells could either be *via* direct binding of metabolites to Treg or *via* changes in cytokines profiles in DCs, which take up and process these metabolites.

### Glucose

Glucose is a critical fuel for Treg cell ATP generation, cell activation, and function. Glucose transporter-1 (Glut-1) levels are low in Treg cells compared with effector cells because FoxP3 limits Glut-1 expression through inhibition of Akt (36). Treg cells exhibit low to modest glycolysis compared with effector T cells along with elevated mTOR activity (37–39). Kishore and colleagues recently demonstrated that enzyme glucokinase (GCK)-dependent glycolysis regulates Treg-cell migration as GCK promotes cytoskeletal rearrangements by associating with actin. Treg cells lacking this pathway were functionally suppressive but failed to migrate to skin allografts and inhibit rejection (40).

### Fatty Acids

The colonic microbiota metabolizes complex carbohydrates and undigested dietary fibers to oligosaccharides and monosaccharaides, which are then fermented to short-chain fatty acids (SCFAs); acetate, propionate, and butyrate (41). Free FAs can diffuse across the plasma membrane into the cytosol. FAs are categorized into groups based on the length of their aliphatic chain. SCFAs have 2–6 carbons; medium chain fatty acids (MCFAs) have 7–12 carbons; and long-chain fatty acids (LCFAs) have more than 12 carbons. SCFAs and MCFAs are absorbed directly into the blood *via* intestinal capillaries and travel through the portal vein. However, LCFAs are absorbed into the intestinal villi and reassembled again into triglycerides. The triglycerides are coated with cholesterol and protein (protein coat), forming chylomicron, which is carried *via* the lymphatics to drain into the systemic circulation (Figure 2). MCFAs and LCFAs are considered one of the most abundant components of the “Western diet” (42). The concentration of SCFAs is highest in the proximal colon where fermentation mostly occurs.

### Short-Chain Fatty Acids

Short-chain fatty acids exert metabolic regulation by signaling through metabolite- sensing G-protein-coupled receptors (GPCRs). GPCR43, or free fatty acid receptor 2, binds to SFCAs (43) (Figure 1). Immune cells such as Treg cells and DCs express GPR43, which bind SCFAs and promote their differentiation and function to maintain intestinal homeostasis (43). Regulation of colonic and pTreg-cell numbers also relies upon the expression of GPR103, a receptor for Niacin (vitamin B3), which is expressed on DCs, which promote Treg-cell differentiation (44, 45) (Figure 2). The SCFA, butyrate has been shown to inhibit histone deacetylase (HDAC) thereby enhancing histone acetylation in the FoxP3 promoter region to promote stable FoxP3 expression (45). Butyrate also promotes the extra-thymic induction of Treg cells *via* the intronic enhancer conserved non-coding sequence 1 (46).

GPCR84 recognizes MCFAs. LCFAs are transported across the membrane by fatty acid translocase (or CD36) (47) or GPCR40 (free FA receptor-1) (48) (Figure 1). The effect of LCFAs was studied recently by Haghikia and colleagues in EAE reporting that the addition of lauric acid (C12), to a culture of CD4^+^ T cells, not only increases the differentiation of Th1 and Th17 cells but also leads to a reduction in Treg cells (49).

Data on immunometabolism on tissue resident T cells are limited. Recent data from Pan and colleagues described that mouse CD8 tissue resident memory cells generated by viral infection of the skin differentially express high levels of molecules that mediate lipid such as fatty acid-binding proteins 4 and 5. They then continued to link this finding with human psoriatic skin suggesting the important role of FAs and their oxidative metabolism for tissue resident cells to mediate protective immunity (50).

Investigating the role of SCFAs on human T cells is in its early stage. Peripheral blood mononuclear cells of healthy donors exposed to SCFAs, especially butyrate, reduced IL-6 levels and hence increased the differentiation of Treg cells over Th17 cells (51). Indeed, this is supported by the recent data from Schmidt et al. suggesting that exposure to butyrate along with TGF-β1 enhanced Foxp3 induction in human T cells to a greater extent than TGF-β1 alone (52).

### Amino Acids

Amino acids and peptides are generated in the gut *via* the hydrolysis of endogenous and alimentary proteins by extracellular proteases and peptidases derived from the pancreatic and other digestive enzymes and commensal bacteria residing in the gut. Amino acids can serve as sources for metabolites that enter into the metabolic tricarboxylic acid (TCA) cycle. In the context of T cell biology, arginine (Arg), glutamine (Glu), and Trp are critical for efficient T cell function and proliferative responses.

### Arg and Treg-Cell Proliferation

Metabolic activity is intimately linked to T cell function. Arg is transported to the cells by cationic transporters CAT1–4 (53) (Figure 1). BAZ1B, PSIP1, and Translin are also potential Arg sensors that promote T cell survival (54). Intracellular Arg is metabolized by arginase 1 and nitric-oxide synthase 2. The human liver contains myeloid-derived suppressor cells, which consume and deplete extracellular Arg (55). Arg-depleted environments impair T cell proliferation.

### Glu and Treg Survival, Proliferation

The amino acids leucine and Glu enter into T cells *via* their transporter LAT1 (CD98 for leucine and ASCT2 for Glu) (Figure 1). Glu-deprived activated CD4 T cells differentiate into Treg cells rather than Th1 cells even in the presence of cytokines that would normally favor Th1 cell differentiation (56). Treg cells do not require LAT1 or ASCT2 for their differentiation *in vitro* (57). Treg-cell differentiation is favored during Glu deprivation (56) *via* mTOR signaling (58). Both leucine and Glu are positive regulators of CD4 T cell differentiation into Th1 and Th17 cells because absence of LAT1 expression impairs the differentiation of these lineages (59), and elevated Glu levels favor Th1 and Th17 cell differentiation (57). Glu-derived metabolite α-ketoglutarate promotes Th1 differentiation through enhancing mTORC1 signaling (56).

### Trp and Treg-Cell Function

Dietary aryl hydrocarbon receptor (AhR) ligands absorbed *via* the gut have been shown to be involved in Th17 generation (60, 61). Trp, an essential amino acid derived from ingested proteins, is one of the AhR ligands. On the other hand, IDO in DCs catabolizes Trp, resulting in localized Trp depletion. Trp-derived kyurenins are a crucial mechanism of Treg-cell suppression (62) (Figure 2). Thus, different metabolites at different stages have their own roles in T cell lineage differentiation.

### Vitamins and Treg Function

Treg-cell biology depends on vitamins A, D, B3 (Niacin), and B9. Fat-soluble vitamins, vitamin A, D, E, and K, are enriched in the human liver (Figure 2). Vitamin A, RA, has an important role in Treg-cell development and function in the gut *via* CD103 DCs in the mesenteric lymph nodes (63). RA can also generate gut homing Treg cells (64). Vitamin B3, nicotinic acid, signals through GPCR109a and leads to expression of retinal dehydrogenases in colonic DCs, which in turn induces Treg-cell differentiation thus vitamin B3 promotes colonic Treg-cell generation (65). It is likely that these DC subsets may be present in the inflamed human liver *via* the portal vein (Figure 2). 1,25(OH)_2_ vitamin D3 inhibits T effector cell proliferation, induces Foxp3 expression, and enhances the suppressive activity of Treg cells (66). Folic acid, derived from vitamin B9, is required for DNA synthesis and repair. Human Treg cells express high level of folate receptor-4 (FR4) (67) and inhibit Treg-cell apoptosis (68) (Figure 2).

### T Cell Migration and Metabolism

The majority of liver resident immune cells, including Treg cells, are normally observed within the hepatic portal tract and septum (interface hepatitis) and parenchyma (lobular hepatitis) depending on the local area of inflammatory response (69). We reported previously that the CXCR3–CXCL10 pathway is crucial for recruitment of blood Treg cells to the inflamed liver *via* the hepatic sinusoids (70) and the CCR6–CCL20 axis plays an essential role in the positioning of lymphocytes around the bile ducts (71). We have also shown that the survival of intrahepatic lymphocytes depends on VCAM1 expression on the bile ducts and VLA-4 expression on the lymphocytes (72). Once circulatory Treg cells are recruited *via* the sinusoids, their post-endothelial migration and positioning are influenced by integrin expression on the fibrous stromal framework in the liver and also the chemokines gradient (73). Some intrahepatic Treg cells may drain back to local draining portal lymph nodes, which drain the liver. In addition, the hepatic microenvironment is hypoxic, especially around the central vein region and there is a high level of lactate in the inflamed human liver. T effector cell migration is known to be highly dependent on aerobic glycolysis, and lactate seems to regulate their migration (74). Recent data suggested that glycolysis was instrumental for Treg migration and was initiated by pro-migratory stimuli *via* a PI3K–mTORC2-mediated pathway culminating in induction of the enzyme GCK. Subsequently, GCK promoted cytoskeletal rearrangements by associating with actin. Treg cells lacking this pathway were functionally suppressive but failed to migrate to skin allografts and inhibit rejection suggesting that GCK-dependent glycolysis regulates Treg-cell migration (40).

## Epigenetic Control of T Cell Metabolism

Epigenetic mechanisms, such as histone modification, DNA methylation of CpG residues, and nucleosome repositioning, alter the accessibility of transcription factors and RNA polymerase to regulatory regions of the genome are important regulators of the immune cells and their metabolism.

### Methylation

The stable expression of Foxp3, which is important for Treg cell’s suppressive function, is maintained *via* the demethylation of the TSDR (75, 76). However, pTreg cells show a lack of Treg-specific DNA hypomethylation, which correlates with Treg cell’s genetic signature (75). Stable Foxp3 requires DNA hypomethylation at FOXP3 CNS2 (76, 77).

### Acetylation, HDAC Inhibitor, and Metabolism

**Figure 3:**
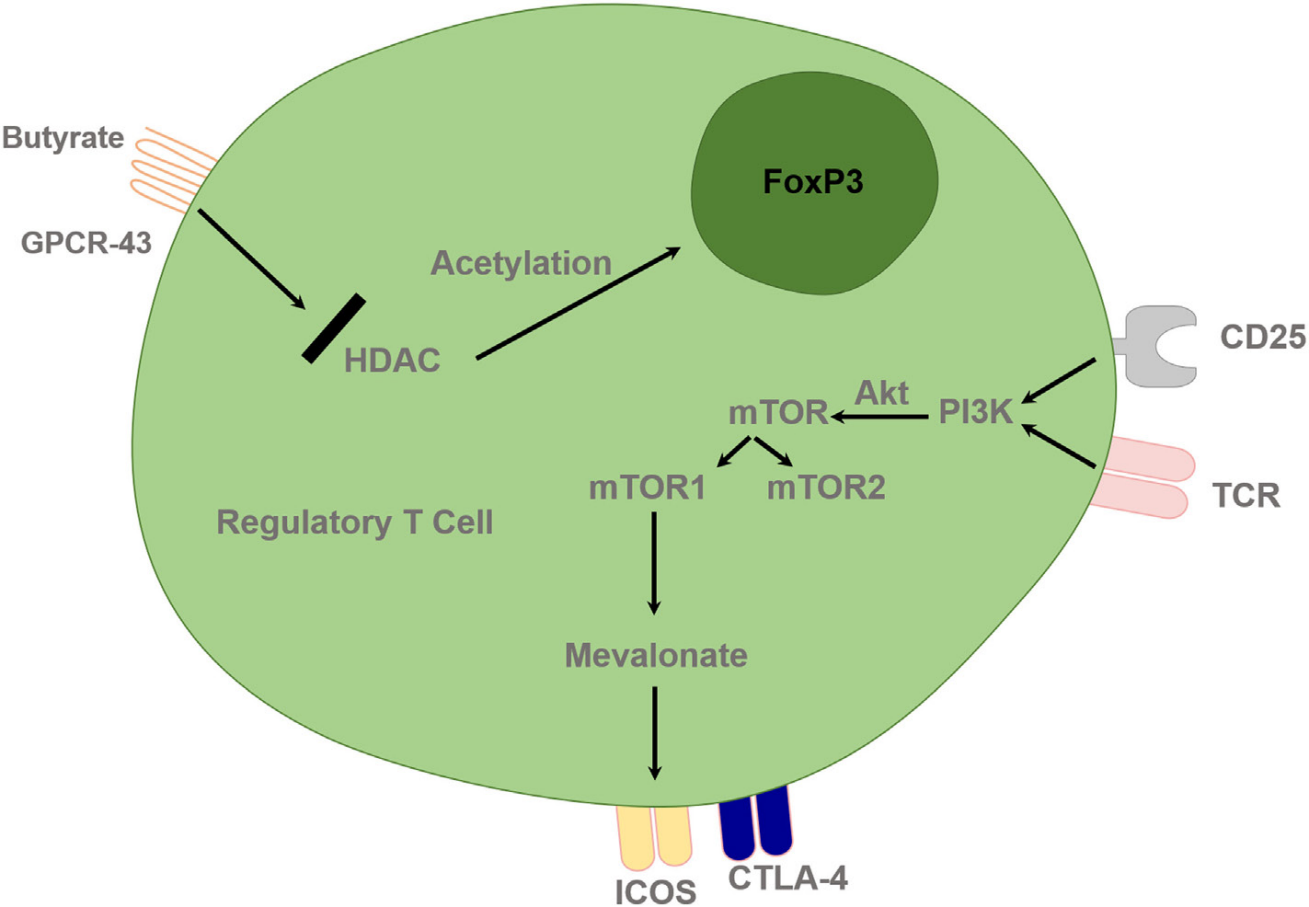
Regulatory T cells are controlled by epigenetic mechanisms and T cell receptor (TCR) and interleukin-2 (IL-2) pathway. Histone acetylation facilitates FoxP3 gene expression and deacetylation inhibits gene expression. HDACi, such as butyrate, can lead to upregulation of the Treg-cell master regulatory gene, FoxP3, and enhance its function. TCR and IL-2 combined stimulation leads to PI3 kinase activation and subsequent downstream activation of mTOR signaling *via* Akt. Treg cells require lipogenic metabolism *via* the mevalonate pathway, which subsequently leads to upregulation of Treg-cell suppressive molecules cytotoxic T lymphocyte-associated antigen 4 (CTLA-4) and ICOS in mTORC1-dependent manner. HDAC, Histone deacetylase inhibitor; PI3K, phosphatidylinositol 3-kinase; Akt, Protein Kinase B; mTOR, mammalian target of rapamycin; GPCR, G protein–coupled receptor.

Epigenetic modifications influence the chromatin remodeling *via* acetylation; DNA methylation and histone modifications play a key role in the regulation of metabolic gene expression and cell differentiation, function, and recruitment. Histone acetylation by histone acetyl transferases allows gene expression and histone deacetylation by HDACs, which inhibits gene expression as well as regulating chromatin remodeling and functional transcription factors. Administration of an HDAC inhibitor (HDACi) *in vivo* increased Foxp3 gene expression, as well as the production and suppressive function of Treg cells. HDAC9 seems to be particularly important in regulating Foxp3-dependent suppression. HDACi therapy *in vivo* enhanced Treg cell-mediated suppression and decreased the degree of inflammatory bowel disease (78). Thus, pharmacological inhibitors of HDAC have potential therapeutic benefits in autoimmunity. Their action may also be mediated *via* immunometabolism; for example, HDAC inhibition leads to the induction Treg-cell generation by butyrate (78). HDACis, such as trichostatin A, SAHA, butyrate, and valproic acid, lead to immunomodulation by upregulating Treg-cell programming and suppressing Th1/Th17 programming (79) (Figure 3).

## Translational Immunometabolism of Regulatory T Cells

Manipulation of metabolism to enhance Treg-cell function in autoimmune diseases is an exciting therapeutic avenue. Dysregulated effector T cell responses and failure of T regulatory cell suppression of these effector cells is a typical feature of autoimmunity. Repurposing of current metabolic drugs and exploring new targets to change immune balance is currently an attractive translational option for clinicians and scientists who aim to apply cell metabolism for patient benefit.

### Targeting Carbohydrate Metabolism

Although it is accepted that repression of Akt/mTOR, hypoxic induced factor 1-α (HIF-1α), and aerobic glycolysis is important for the efficient generation of blood Treg cells *in vitro*, clear evidence how these pathways impact on blood and tissue resident Treg-cell development *in vivo* is limited. Understanding these pathways, not only in the circulation but also at the tissue level is necessary to enhance Treg-cell metabolism and its subsequent function. Although Glut-1 expression is a critical factor in driving glycolysis in circulatory T effector cells, Treg cells’ function seems to be independently of Glut-1 (80).

#### HIF-1α Pathway

The hepatic environment in both inflammatory and autoimmune conditions is hypoxic as human liver receive only 25% of oxygen rich blood supply from hepatic artery, and the rest of the blood supply is from portal vein (1). Thus, targeting the transcription factor HIF-1α is a promising strategy as it exerts a crucial role in the balance between Th17 and Treg cells (81). HIF-1α predominantly affects effector T cell metabolism compared with Treg, thus there is a shift in immune cells balance under hypoxic conditions (82). HIF-1α and aryl hydrocarbon receptor (AhR) compete for limited amounts of aryl hydrocarbon receptor nuclear translocator (ARNT) also know as HIF-1β. This competition is the key to the mutual regulation of HIF-1α and AhR (83) (Figure 1). ARNT serves as a common binding partner for AhR as well as HIF-1α. HIF-1α proteins are regulated in an oxygen-dependent manner, whereas ARNT is constitutively expressed, as neither ARNT mRNA nor the protein level is influenced by hypoxia. In the context of transcription factor, HIF-1α induces FoxP3, which leads to Treg-cell abundance, and Treg-intrinsic HIF-1α is required for optimal Treg function (84). HIF-1α-deficient Treg cells fail to control T-cell-mediated colitis (84).

HIF-1α is selectively expressed in Th17 cells, and its induction requires signaling through mTOR, a central regulator of glycolytic metabolism. Therefore, blocking glycolysis inhibited Th17 cell development while it promotes Treg-cell generation (39). Lack of HIF-1α *in vivo* has been reported to diminish Th17 cell development but enhance Treg-cell differentiation and prevent autoimmune neuroinflammation (39).

#### Metformin and 2-Deoxy-d-Glucose (2DG)

We reported that human liver infiltrating Treg cells are of an effector memory phenotype (16). However, intrahepatic Treg metabolic phenotype is still unexplored. When resting naive T cells are activated, they differentiate toward an effector T cell lineage due to a shift in the catabolic state of metabolism, which is driven predominantly by the glycolytic–lipogenic pathway through the TCA cycle. This upregulation of aerobic glycolysis, “Warburg effect,” is a feature of activated immune cells including T cells and is dependent on an mTOR–nutrient-sensing pathway with signaling *via* phosphoinositide 3-kinase (PI3K) and protein kinase B (Akt) (85). In addition, CD4 T cell differentiation into the effector T cell lineage toward Th1 or Th17 cell phenotypes is dependent on glucose and Glu for anabolic metabolism. Recent study demonstrated that CD4 effector T cells from lupus-affected mice showed elevated glycolysis and mitochondrial oxidative metabolism and inhibition of these pathways with *mitochondrial metabolism inhibitor metformin and the glucose metabolism inhibitor 2DG* reduced IFNγ production (86). Furthermore, blocking glycolysis *via* 2DG can selectively impair effector T cells thereby by improving Treg-cell function in a mouse model of multiple sclerosis (87). Antidiabetic medication, Metformin not only reduce Th17 cell responses and attenuates disease severity in experimental autoimmune encephalomyelitis (88) but also increase numbers of Treg cells *via* suppressing the activation of mTOR and its downstream target, HIF-1α (89). Thus, priming the T cells with Metformin may be an attractive option to shift the immune cell balance to the regulatory arm.

#### Soraphen A

Th17 cells, but not Treg cells, depend on ACC1 (acetyl CoA carboxylase 1), a key enzyme that drives FA synthesis and the underlying glycolytic–lipogenic metabolic pathway for their development. Treatment with the ACC-specific inhibitor Soraphen A or T cell-specific deletion of ACC1 in mice attenuates Th17 cell-mediated autoimmune disease (90). Although Th17 cells use this pathway to produce phospholipids for cellular membranes, Treg cells readily take up exogenous FAs for this purpose. Pharmacologic inhibition or T cell-specific deletion of ACC1 not only blocks *de novo* FA synthesis but also interferes with the metabolic flux of glucose-derived carbon *via* glycolysis and the TCA cycle. Thus, the ACC1 pathway could be an attractive option to alter immune cell balance.

#### Phosphatase and Tensin Homolog (PTEN)

Phosphatase and tensin homolog lipid phosphatase is the main negative regulator of PI3K–Akt signaling and glycolysis in Treg cells. The activity of phosphoinositide-3-kinase (PI3K) is essential for Treg-cell lineage homeostasis and stability. Mechanistically, PTEN maintained Treg-cell stability and metabolic balance between glycolysis and mitochondrial fitness (91). Control of PI3K signaling by PTEN in Treg cells is critical for maintaining their homeostasis, function, and stability (92). PTEN deficiency upregulates activity of the metabolic checkpoint kinase complex mTORC2, and the serine–threonine kinase Akt, and loss of this activity restores functioning of PTEN-deficient Treg cells. Thus, PTEN–mTORC2 axis maintains Treg-cell stability and coordinates Treg cell-mediated control of effector responses (91), and PTEN inhibitor can lead to Treg destabilization (93).

### Targeting Lipid Metabolism

#### Mevalonate and Statins

Cholesterol lowering medications, statins are inhibitors of the enzyme 3-hydroxy-3-methylglutaryl coenzyme A reductase, which catalyzes the formation of mevalonate, the rate-limiting step for cholesterol synthesis. As a result, statins are widely used for cardiovascular disease prevention. Statins can differentiate T cells toward Treg cells instead of Th17 cells *via* a mechanism dependent on protein granulation (94). In general, Treg cells require lipid and cholesterol metabolism. The mevalonate pathway is particularly important for coordinating Treg proliferation and for upregulating the suppressive molecules CTLA-4 and ICOS to establish functional competency of Treg. Mevalonate can reverse the effects of statins involved in maintaining Treg functional fitness in an mTORC1-dependent manner (37) (Figure 3). Thus, mevalonate pathway could be manipulated to enhance Treg function in autoimmunity.

However, recent work from Hu and colleagues seems to contradict this finding as they suggested that cholesterol biosynthesis and uptake programs are induced during Th17 differentiation, resulting in the accumulation of the cholesterol precursor, desmosterol, which functions as a potent endogenous RORγ agonist (95, 96). Thus, blocking cholesterol synthesis with chemical inhibitors at steps before the formation of active precursors would reduce differentiation to Th17. In addition, Simvastatin has also been shown to improve disease activity and the inflammation factor in patients with multiple sclerosis (97) and SLE (98). As there are conflicting data, more studies are required to dissect the mechanistic immunomodulatory effects of statins on Treg and Th17 cells.

#### Rapamycin and mTOR

The activation of mTOR, which is the catalytic subunit of the mTORC1 and mTORC2 complexes, delivers signals for the activation and differentiation of effector CD4 T cells, whereas Akt–mTOR axis is a crucial negative regulator of Treg *de novo* differentiation and expansion (Figure 3). mTORC1 signals through the TCR and the co-receptor CD28, and Treg cells have elevated steady-state mTORC1 activity compared with naive T cells. Signals through the TCR and IL-2 provide major inputs for mTORC1 activation, which in turn programs the suppressive function of Treg (Figure 3). Disruption of mTORC1 through Treg-specific deletion of the essential component raptor results in profound loss of Treg-cell suppressive activity and the development of a fatal early onset inflammatory disorder (37). In addition, Raptor–mTORC1 signaling in Treg cells promotes cholesterol and lipid metabolism, with the mevalonate pathway particularly important for coordinating Treg-cell proliferation and upregulation of the suppressive molecules CTLA-4 and ICOS to establish Treg functional competency (Figure 3). Thus, mTORC1 acts as a “rheostat” in Treg cells to link immunological signals from TCR and IL-2 to lipogenic pathways and functional fitness, and highlights a central role of metabolic programming of Treg suppressive activity in immune homeostasis and tolerance. All these data may lead investigators to reconsider and dissect the current use of rapamycin in good manufacturing practice (GMP) Treg-cell culture media (to prevent effector Th17 cells outgrowth) in Treg-cell therapy setting.

#### Peroxisome Proliferator-Activated Receptor (PPAR) Agonist—Pioglitazone

Peroxisome proliferator-activated receptors are nuclear receptors that regulate gene transcription. PPARα is highly expressed in liver and skeletal muscle and controls genes involved in fatty-acid oxidation (99). PPARγ is expressed in adipocytes, skeletal muscle, liver, and kidney and regulates expression of the genes that mediate metabolism (100). PPARγ agonists, thiazolidinedione drug pioglitazone, could potentially become an attractive drug candidate for anti-inflammatory therapies.

### Targeting Protein Metabolism

#### Glu and α Ketoglutarate

Glutamine (Glu), a central anabolic nutrient in the TCA cycle, is critical for T cell survival, proliferation, and function. Glu is required for naive CD4 T cell differentiation toward Th1 and Th17 inflammatory T cells. In patients with multiple sclerosis, increased levels of both Glu and glutamate have been reported (101, 102). TCR engagement of naive CD4 T cells has been shown to trigger rapid uptake of Glu, *via* amino acid transporters. Glu deprivation has been shown to enhance the suppressive activity of Treg cells in an autoimmune colitis model (56). Thus, decline in Glu and α-ketoglutarate, Glu-derived TCA cycle metabolite could enhance Treg cells’ function.

## Benefits and Shortcomings of Current Technology

Both glycolysis and mitochondrial respiration can be studied using a Seahorse machine for immune cell subsets including peripheral blood CD4 and CD8 T cells. Extracellular acidification rate (ECAR), a measure of lactate production by glycolysis, and mitochondrial oxygen consumption rate of both blood and tissue resident cells are necessary to analyze the metabolism and function of these cells. However, Seahorse technology is still not possible to apply for small frequency cell subset, such as regulatory T cells, to perform ECAR and OCAR experiments without cell expansion. However, expansion of the Treg will change their metabolic phenotype. Similarly, metabolic tracing with fluorescence uptake of glucose, Glu, lactate, or palmitate of Treg requires a significant number of cells. Thus, current available methodology to study tissue derived Treg is limited to Mitotracker and TMRE assays along with electron microscopy. In addition, comparing the metabolic activity of glycolysis and mitochondrial respiration in central memory (CD45RA^−^CCR7^+^), naïve (CD45RA^+^CCR7^+^), tissue resident (CD45RA^+^CCR7^−^), and effector memory (CD45RA^−^CCR7^−^) subsets of intrahepatic Treg cells is crucial to understand the metabolism and functional potential of each subset. However, investigators are limited to perform these analyses not by Seahorse technology but only by Mitotracker and TMRE assays along with electron microscopy due to the current requirement of high cell numbers. In addition, studies to assess tissue resident cell metabolism under hypoxic conditions and their migration would require a modified combined technology of hypoxic chambers or migration chambers in combination with Seahorse equipment.

## Conclusion

T cell metabolism and immunology have recently been merged to form immunometabolism. Intrahepatic Treg cells can control local hepatic immune homeostasis. There is enormous potential to utilize Treg to restore tolerance in the treatment of human autoimmune diseases including autoimmune liver diseases. Modulation of immunometabolism of Treg represents a new avenue to enhance Treg-cell function and maintain a stable lineage. Immunometabolic manipulation may also have an impact on Treg cytoskeletal rearrangement and post-endothelial migration, positioning around hepatocytes and bile ducts and retention as intrahepatic tissue resident Treg. However, to date, there are no data on human liver tissue resident Treg-cell metabolism. In the future, improvement of technology may allow us to study the metabolic profile and associated function Treg. Manipulating the cell culture media to enhance the metabolism of Treg during GMP isolation and expansion and modulating the tissue and circulatory compartments with immunometabolic drugs in autoimmune patients before Treg infusion would enhance the potential of effective and successful Treg-cell therapy.

## Author Contributions

RW and HB wrote the manuscript. RW and YO constructed the mechanistic Figures 1–3. YO supervised and edited the final manuscript.

## Conflict of Interest Statement

The authors declare that the research was conducted in the absence of any commercial or financial relationships that could be construed as a potential conflict of interest.
